# FCGR3A: A new biomarker with potential prognostic value for prostate cancer

**DOI:** 10.3389/fonc.2022.1014888

**Published:** 2022-11-24

**Authors:** Zeyu Zha, Yuan Hong, ZhenFeng Tang, Qiuling Du, Yan Wang, Shengbang Yang, Yongding Wu, Huijing Tan, Funneng Jiang, Weide Zhong

**Affiliations:** ^1^ School of Medicine, Jinan University, Guangzhou, China; ^2^ The Second Affiliated Hospital of Bengbu Medical College, Bengbu, China; ^3^ Department of Urology, Guangdong Key Laboratory of Clinical Molecular Medicine and Diagnostics, Guangzhou First People’s Hospital, School of Medicine, South China University of Technology, Guangzhou, China; ^4^ College of The First Clinical Medicine, Guangzhou University of Chinese Medicine, Guangzhou, China; ^5^ School of Medicine, Guizhou University, Guiyang, China

**Keywords:** prostate cancer, prognosis, FCGR3A, tumor microenvironment, bioinformatics analysis

## Abstract

To screen target gene cluster by bioinformatics analysis and verify them by *in vitro* experiment and clinicopathological correlation analysis. We try to find a new biomarker with prognostic value for prostate cancer (PCa). 42 candidate marker genes were constructed by protein protein interaction (PPI) network and enriched by KEGG pathway to find out the gene cluster we are interested in. Prognostic model was established to preliminarily analyze the prognostic value of this gene cluster in PCa, and Cox risk regression was used for comparative analysis. Immunohistochemistry was used to detect the expression of each gene in clinical tissue microarray. Finally, we analyzed the correlation between each gene and their clinicopathological features of PCa combined with TCGA clinical data. Based on the analysis of PPI and KEGG, we found the target gene cluster (FCGR3A, HAVCR2, CCR7 and CD28). Prognostic model analysis showed that this gene cluster had the ability to predict biochemical recurrence, and the survival rate and ROC analysis showed favorable prediction effect. Univariate Cox regression analysis showed that the risk scores of Gleason score (GS), T stage, N stage and PSA were significantly different (*P*<0.05), and the risk ratio of high expression was 2.30 times that of low expression (*P*=0.004). However, it was not statistically significant in multivariate Cox regression analysis (*P*>0.05). The results of tissue microarray showed that FCGR3A and HAVCR2 were highly expressed in PCa (*P*<0.01), while the expression of CCR7 and CD28 had no significant difference (*P*>0.05). Kaplan-Meier analysis showed that there was significant difference in BCR free survival of FCGR3A and HAVCR2 (FCGR3A, *P*=0.010; HAVCR2, *P*=0.018), while the expression of CCR7 and CD28 had no significant difference on the survival and prognosis of PCa patients (*P*>0.05). TCGA clinical data analysis found that the expression of FCGR3A had a unique correlation with the clinicopathological features of PCa, which was closely related to the tumor stage. The expression of FCGR3A is related to BCR free survival of PCa patients. Therefore, FCGR3A is a new biomarker with potential prognostic value of PCa.

## Introduction

Prostate cancer (PCa) is a malignant tumor and is the second most fatal cancer for men (1). Due to an aging population, the morbidity of PCa is increasing (2). As the early symptoms of PCa are not obvious, most PCa patients are in the terminal stage when they are examined and then with short survival period (3, 4). Although the current clinical treatment, including enzalutamide and abiraterone therapy, can significantly improve the overall survival rate of PCa patients, is not ideal for PCa patients with terminal stage (5–7). Prostate specific antigen (PSA) is a biomarker of PCa, but its detection results are easily affected by drugs, inflammation and benign prostate lesions, especially lack of specificity and sensitivity in the early diagnosis and prediction of recurrence of PCa (8, 9). Therefore, it is necessary to study the progression of PCa and identify the operative prognosis biomarkers.

Tumor microenvironment (TME) is composed of different cell subsets, including tumor cells, blood vessels, immune cells, stromal cells and other ingredients (10). PCa is highly heterogeneous in TME, so the morbidity of terminal or metastatic PCa (especially bone metastasis) has increased over the last years (11–13). However, whether the changes of cell subsets in TME are related to tumor prognosis has aroused our intense concern. In a recent study (14), Researchers obtained 36424 cells from 13 prostate tumor tissues and performed single cell transcriptome sequencing on these cells and found that multiple progression-related transcriptome programs were activated in TME and 42 marker genes (ACTA2, PECAM1, VWF, ENG, CMA1, MS4A2, TPSAB1, TPSB2, AR, KRT19, KRT18, KRT8, TP63, KRT14, KRT5, LYZ, FCGR3A, CSF1R, CD68, CD163, CD14, UCHL1, HAVCR2, PDCD1, CTLA4, CD8A, SELL, PTPRC, CD4, BTLA, IL2RA, IL7R, CCR7, CD28, CD27, SLAMF1, DPP4, CD7, CD2, CD3G, CD3E and CD3D) were identified in multiple cell subpopulations including T cells, monocytes, endothelial cells and fibroblasts. We concluded that these genes are mainly related to cell communication, antigen presentation process and immune cell receptor signaling pathway. And some of them have been predicted to have favorable prognostic value in PCa, such as VWF, AR and CSF1R (15–18). The above 42 marker genes are selected as candidate genes for bioinformatics analysis and we attempt to discover a new biomarker with potential prognostic value of PCa.

FCGR3A (Fc fragment of IgG receptor IIIa), a transmembrane glycoprotein, is expressed on natural killer cells and inhibits the growth of liver tumor cells. FCGR3A gene is associated with the risk of lesions in several oncological diseases, for example, FCGR3A gene polymorphisms are positively associated with the risk of lesions in colorectal cancer, and genetic variants of FCGR3A are associated with drug resistance in rheumatoid arthritis (19, 20). FCGR3A has been shown to be involved in multiple immune cell infiltration and DNA mismatch repair genes, while drug sensitivity analysis showed that higher FCGR3A expression predicted lower IC50 (half maximal inhibitory concentration) values for the majority of drugs (21), however, the prognostic value of FCGR3A expression in prostate tumors and its correlation with immune infiltration are unclear.

In recent decades, the advancement of bioinformatics has enabled researchers to more comprehensively investigate its biological mechanisms and more effectively identify key therapeutic and prognostic molecules. In this study, we performed a series of comprehensive bioinformatics analyses of tumor-associated genes. Our study aims to provide new therapeutic targets for prostate cancer and help to understand the underlying immune mechanism of prostate cancer. At the same time, through the analysis of various cancer-related genes, we can understand the development correlation of various diseases and provide a basis for combined treatment.

## Materials and methods

### PPI network construction

Protein-protein interaction database, String (https://cn.string-db.org/), was used to calculate and analyze the interaction relationship between 42 candidate genes. Cytoscape software (V3.8.2) was used to visualize the gene interaction network.

### KEGG pathway enrichment analysis

KEGG rest API (https://www.kegg.jp/kegg/rest/keggapi.html) was used for the latest gene annotation as background. Then, 42 candidate genes were mapped into the background set, and R software package, clusterProfiler (Version 3.14.3), was used for enrichment analysis to obtain gene enrichment results. Set the minimum gene set as 5 and the maximum gene set as 5,000, *P*<0.05 and *FDR*<0.1 was considered statistically significant.

### Prognostic model construction

PCa data used in this study downloaded from the public database TCGA (https://xenabrowser.net/). The downloaded data type is count, and Deseq2 is used to standardize the data. The clinical information of PCa sample downloaded from cBioPortal (http://www.cbioportal.org/). We constructed prognostic models for the target gene cluster (FCGR3A, HAVCR2, CCR7 and CD28). Prognostic model predicts the prognosis of patients according to the level of risk score by calculating it in each sample. The expression data used in this analysis downloaded from TCGA’s PRAD dataset (PRAD had 427 samples with both RNA SEQ data and BCR data).

The samples of PRAD were divided into training set (214 samples) and test set (213 samples) according to 1:1. First, the risk score of the training set samples was sorted from small to large, and the samples were divided into low-risk group (n=107) and high-risk group (n=107) according to the median of 2.5734. Then, the risk score of the test set samples was sorted from small to large, and the samples were divided into low-risk group (n=117) and high-risk group (n=96) according to the same threshold value as the training set. Finally, integrated the training set and test set samples. The GGlpot2 package of R language was used to make the risk score, survival state and characteristic gene expression diagram. The survminer package was used to make survival curve. The survival ROC package was used to make time-dependent curve. The survival package was used to do univariate Cox regression analysis. The glmnet package was used to do Lasso regression analysis.

### Patients and tissue samples

Tissue microarray (Cat No: DC-PRO01026; TMA, n=80) was purchased from Avilabio Biotechnology Company (Shaanxi Province, China), including 10 prostate samples from healthy individuals and 70 prostate samples from patients with primary PCa, and with information on pathological grade, Gleason grade, Gleason score, TNM and clinical stage. Patients treated with chemotherapy or radiotherapy before the surgery were excluded from this study. In order to quantify the mRNA expression of FCGR3A, havcr2, CCR7 and CD28, clinical information and gene expression data of 427 PCa patients were collected from TCGA database.

### Immunohistochemistry analysis

Paraffin sections were dewaxed by xylene, soaked in 100%, 95%, 70% ethanol and distilled water for 5 min successively, and then washed with PBS solution. Sections were added with EDTA buffer for microwave antigen repair. Endogenous peroxidase was blocked by incubation in 3% hydrogen peroxide solution at 24°C for 10 min. Antigen was blocked with 5%BSA and incubated at 24°C for 20 min. After sealing, the sections were incubated at 4°C overnight with anti-FCGR3A (rabbit monoclonal antibody, 1:50, ET7109-97, HuaBio), anti-HAVCR2 (mouse monoclonal antibody, 1:800, EM1701-18, HuaBio), anti-CCR7 (rabbit monoclonal antibody, 1:200, ab253187, Abcam) and anti-CD28 (rabbit polyclonal antibody, bs-1297R, Bioss) antibodies. Then, 150 μl secondary antibody was added for incubation, followed by DAB color rendering and hematoxylin redyeing. The positive cell rate and the degree of staining were scored by scanning imaging. Positive cell rate score: 0%-10%, 1 point; 10%-50%, 2 points; 50%-75%, 3 points; 75%-100%, 4 points. Staining degree score: no positive staining, 0 point; canary yellow, 1 point; brownish yellow, 2 points; tan, 3 points. The immune risk score (IRS) is the product of the above two scores.

### Statistical analysis

Statistical analyzes were performed using SPSS 22.0 software. Continuous variables were expressed as mean ± SD. Kaplan-meier method was used to analyze the relationship between the expression of FCGR3A, HAVCR2, CCR7 and CD28 and the survival period of PCa patients. Pearson’s chi-squared tests and Fisher’s exact test were used to analyze the association of FCGR3A, HAVCR2, CCR7 and CD28 mRNA expression with clinico-pathological features. Student’s T-tests were used to analyze the association of FCGR3A, HAVCR2, CCR7 and CD28 protein expression with clinico-pathological features. Univariate analysis comparisons and multivariate survival comparisons were performed using Cox proportional hazard regression models. Differences were statistically significant when *P*<0.05.

## Results

### Identify target gene cluster: FCGR3A, HAVCR2, CCR7 and CD28

**Figure 1:**
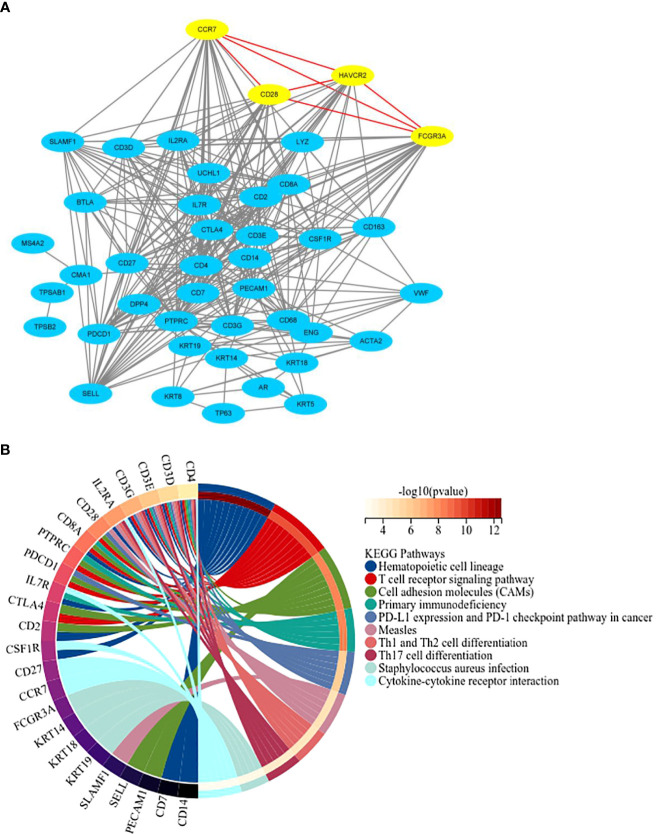
PPI network construction and KEGG pathway enrichment analysis. **(A)** Protein-protein interaction network for 42 candidate marker genes in String database. **(B)** The top 10 pathways of KEGG pathway enrichment analysis.

We constructed PPI networks for 42 candidate genes, and found that FCGR3A, HAVCR2, CCR7 and CD28 interacted with each other (**Figure 1A**), which aroused our curiosity. KEGG pathway enrichment analysis of this gene cluster showed that FCGR3A was mainly enriched in Staphylococcus aureus infection process; CCR7 was mainly enriched in cytokine-cytokine receptor interaction; CD28 was mainly enriched in Hematopoietic cell lineage, T cell receptor signaling pathway, cell adhesion molecules and primary immunodeficiency process (**Figure 1B**). Therefore, we speculate that this gene cluster may be involved in the occurrence and development of PCa. Then, whether this gene cluster can be used as an independent prognostic factor for PCa patients, we will verify our suppose through bioinformatics and *in vitro* experiments.

### Target gene cluster is an independent prognostic factor for the survival of PCa patients

**Figure 2:**
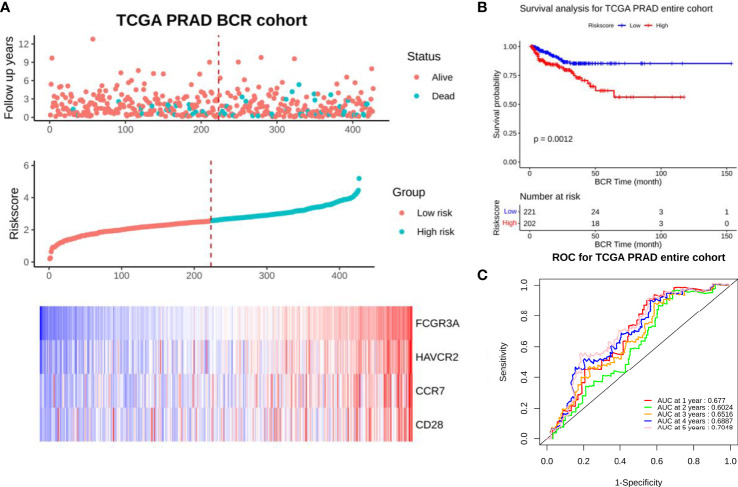
Prognostic model analysis of entire set. **(A)** Distribution of 4 target genes in low-risk and high-risk groups in TCGA-PRAD dataset. **(B)** BCR free survival curve of 4 target genes in TCGA-PRAD dataset (*P*=0.0012). **(C)** 5-year ROC curve of 4 target genes in TCGA-PRAD dataset (AUC>0.6).

We analyzed the relationship between the expression of target gene cluster and the survival of PCa patients by using the TCGA database. After constructing the 4-gene model, we calculated the risk score according to the model of each patient in the cohort and drawn the distribution map. The results showed that the risk of death of high-risk patients (n=96) was significantly higher than that of patients with low risk scores (n=117), and our model could better distinguish the distribution of 4 genes expression in the cohort of low-risk and high-risk PCa patients, among which FCGR3A was the best and CCR7 and CD28 were the worst (**Figure 2A**). Kaplan-meier analysis of survival rate showed that patients with high score had a worse prognosis than those with low score (*P*=0.0012) (**Figure 2B**). ROC analysis showed that the model could effectively predict 5-year survival of PCa patients, and AUC values at 1, 2, 3, 4 and 5 years were 0.677, 0.6024, 0.6516, 0.6887 and 0.7048 respectively (**Figure 2C**). Therefore, the target gene cluster have the ability to predict biochemical recurrence of patients and can be used as independent prognostic factors for the survival of PCa patients.

To further identify whether the expression of target gene cluster can be an independent prognostic factor for PCa patients, we investigated the association between clinical characteristics and risk score. Univariate Cox regression analysis showed that there were significant differences in risk score between tumor and Gleason Score, T stage, N stage and PSA (*P*<0.05), and the risk ratio of high expression was 2.30 times higher than low expression (*P*=0.004) (**Table 1**). Multivariate analysis showed that although there were significant differences in risk score between tumor, T stage and PSA (*P*<0.05), there was no statistical significance in the difference of risk ratio between high expression and low expression (*P*>0.05) (**Table 1**). We hypothesized that this might be caused by the poor distribution of CCR7 and CD28 in the cohort of low-risk and high-risk PCa patients. Subsequently, we verified our suppose by survival analysis of each gene and clinical tissue microarray testing.

**Table 1:** Prognostic value of 4 target genes expression for BCR free survival by Cox proportional hazards model.

Variable	BCR free survival	
	HR (95%CI)	*P*
**Univariate analysis**		
Age (≤60 or >60)	1.10 (0.64-1.90)	0.720
Gleason score (≤8 or >8)	2.70 (1.50-4.60)	<0.001***
Tumor Stage (T1-T2 or T3-T4)	5.20 (2.00-13.00)	<0.001***
Lymph node stage (N0 or N1)	2.00 (1.10-3.50)	0.025*
Distant metastasis (M0 or M1)	0.00 (0.00-2.00)	1.000
PSA (<2 or ≥2)	9.00 (5.00-16.00)	<0.001***
Risk score (Low risk or High risk)	2.30 (1.30-4.20)	0.004**
**Multivariate analysis**		
Gleason score (≤8 or >8)	1.19 (0.66-2.13)	0.568
Tumor Stage (T1-T2 or T3-T4)	3.52 (1.31-9.46)	0.013*
Lymph node stage (N0 or N1)	1.25 (0.68-2.29)	0.474
PSA (<2 or ≥2)	7.19 (3.96-13.04)	<0.001***
Risk score (Low risk or High risk)	1.63 (0.90-2.92)	0.105

＊means the difference is significant at the 0.05 level ,** means the difference is significant at the 0.01 level , *** means the difference is significant at the 0.01 level.

### High expression of FCGR3A and HAVCR2 has a poor prognosis for PCa patients

**Figure 3:**
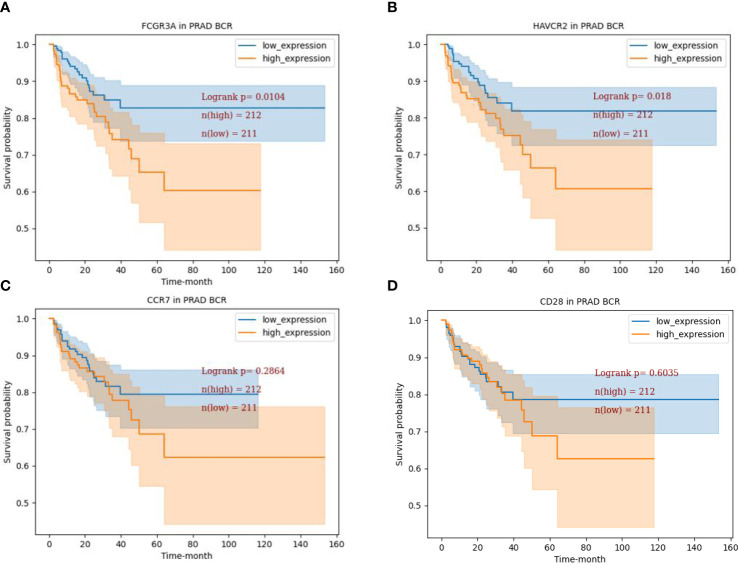
BCR free survival analysis of 4 target genes. **(A)** BCR free survival curve of FCGR3A in TCGA-PRAD dataset (*P*=0.0104). **(B)** BCR free survival curve of HAVCR2 in TCGA-PRAD dataset (*P*=0.0180). **(C)** BCR free survival curve of CCR7 in TCGA-PRAD dataset (*P*=0.2864) **(D)**. BCR free survival curve of CCR7 in TCGA-PRAD dataset (*P*=0.6035).

We used TCGA database to analyze the relationship between the expression of 4 target genes and the survival of PCa patients. The genes expression was divided into high-expression group and low-expression group with median as the boundary. The blue curve (n=211) and the orange curve (n=212) represent the survival of PCa patients with low and high gene expression respectively. Kaplan-meier analysis showed that the BCR free survival of FCGR3A and HAVCR2 was significantly different (FCGR3A, *P*=0.010; HAVCR2, *P*=0.018) (**Figures 3A, B**), indicating that the high expression of FCGR3A and HAVCR2 had a poor prognosis for PCa patients. In addition, the expression of CCR7 and CD28 had no significance for the survival and prognosis of PCa patients (*P*>0.05) (**Figures 3C, D**), indicating that CCR7 and CD28 had no prognostic value.

### High expression of FCGR3A and HAVCR2 in PCa tissues

**Figure 4:**
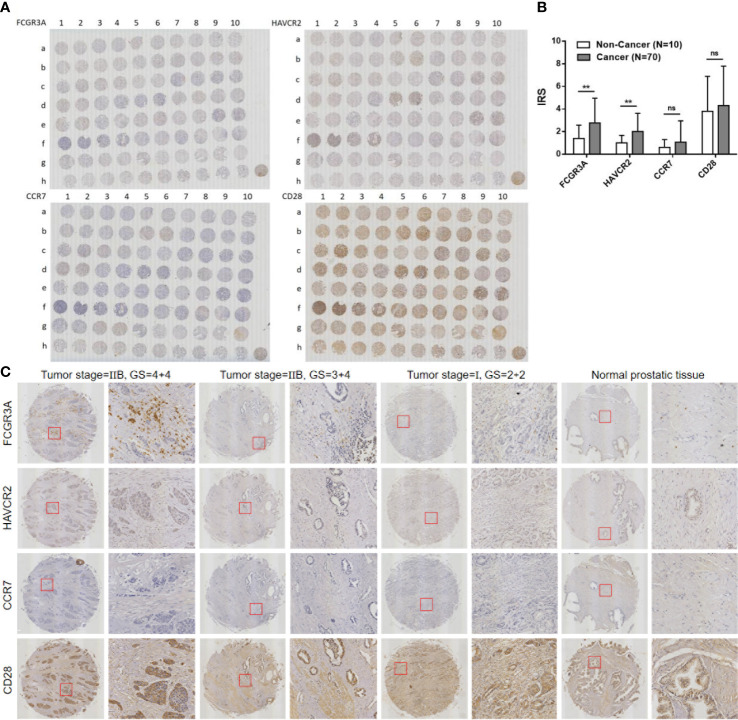
Immunohistochemical staining for 4 target genes expression in PCa and normal prostate tissue samples. **(A)** Full view of the immunohistochemistry staining for 4 target genes expression. **(B)** (no significance, ns P>0.05) Immune risk score (IRS) of 4 target genes in PCa and normal prostate tissue samples (Cancer vs. Non-Cancer, ^**^ *P*<0.01). **(C)** 4 target genes expression in PCa tissue samples with different tumor stages and Gleason score (GS).

We further verified the role of the expression of 4 target genes in the prostate of PCa patients by immunohistochemistry. Immunostaining results showed that FCGR3A, CCR7 and CD28 proteins were all expressed in the cytoplasm and membrane, while HAVCR2 protein was mainly expressed in the cytoplasm and membrane and a small amount in the nucleus (**Figure 4A**). Based on immune response score (IRS=0-4, low expression; IRS=4-12, high expression), we found that FCGR3A and HAVCR2 proteins were up-regulated in prostate tissues of PCa patients compared with normal tissues (*P*<0.01), while the expression of CCR7 and CD28 proteins were not significantly different between them (*P*>0.05) (**Figure 4B**). In addition, we found that the expression of FCGR3A and HAVCR2 proteins was gradually up-regulated with the increase of Gleason score (GS) grade in tissue samples of different tumor development stages (GS=4+4, high; GS=3+4, medium; GS=2+2, low), among which the expression of FCGR3A was most significantly up-regulated, while the expression of CCR7 and CD28 proteins showed no significant difference (**Figure 4C**).

### Expression of FCGR3A is uniquely correlated with the clinicopathological features of PCa

**Tables 2**–**5** summarizes the correlation between the expression of 4 target genes and the different clinicopathological features of PCa patients. In TMA, IRS<4 was considered as low expression, and IR≥4 was considered as high expression. In TCGA, 427 PCa samples were divided into low-expression group (n=213, 49.88%) and high-expression group (n=213, 50.12%). We were surprised to find that FCGR3A expression was inconsistent with clinicopathological features in age and GS, while consistent with clinicopathological features in tumor stage (TMA, *P*=0.030; TCGA, *P*=0.006). TMA and TCGA results showed that FCGR3A was highly expressed in tumor T2 stage (which was consistent with the result of tissue microarray), but showed the opposite result in tumor T3 stage. And the expression of HAVCR2, CCR7 and CD28 had poor correlation with the clinicopathological features of PCa in age, GS and tumor stage.

**Table 2:** Correlation of FCGR3A expression with clinicopathological features in PCa patients.

Clinical features	TMA				TCGA			
	Case	Low, n (%)	High, n (%)	*P*	Case	Low, n (%)	High, n (%)	*P*
**Tissue**								
Cancer	70	32 (45.71)	38 (54.29)	0.397	427	213 (49.88)	214 (50.12)	–
Non-cancer	10	6 (60.00)	4 (40.00)	–	0	–	–	–
**Age (years)**								
≤60	14	7 (50.00)	7 (50.00)	0.837	190	105 (55.26)	85 (44.74)	0.047^*^
>60	66	31 (46.97)	35 (53.03)	–	237	108 (45.57)	129 (54.43)	–
**Gleason score**								
≤8	46	22 (47.83)	24 (52.17)	0.623	300	171 (57.00)	129 (43.00)	<0.001^***^
>8	24	10 (41.67)	14 (58.33)	–	127	42 (33.07)	85 (66.93)	–
**Serum PSA levels (ng/ml)**								
≤2	–	–	–	–	378	196 (51.85)	182 (48.15)	0.003^**^
>2	–	–	–	–	35	9 (25.71)	26 (74.29)	–
**Pathological grade**								
pT1-pT2	4	3 (75.00)	1 (25.00)	0.213	–	–	–	–
pT3	65	28 (43.08)	37 (56.92)	–	–	–	–	–
**Tumor stage**								
T1	0	–	–	**-**	153	92 (60.13)	61 (39.87)	0.006^**^
T2	42	16 (38.10)	26 (61.90)	0.030^*^	155	75 (48.39)	80 (51.61)	–
T3	14	10 (71.43)	4 (28.57)	–	48	16 (33.33)	32 (66.67)	–
T4	0	–	–	–	1	0 (0.00)	1 (100.00)	–
**Lymph nodemetastasis**								
N0	56	26 (46.43)	30 (53.57)	–	303	155 (51.16)	148 (48.84)	<0.001^***^
N1	0	–	–	–	69	20 (28.99)	49 (71.01)	–
**Distant metastasis**								
M0	56	26 (46.43)	30 (53.57)	–	401	200 (49.88)	201 (50.12)	0.568
M1	0	–	–	–	3	1 (33.33)	2 (66.67)	–

＊means the difference is significant at the 0.05 level ,** means the difference is significant at the 0.01 level, *** means the difference is significant at the 0.01 level.

**Table 3:** Correlation of HAVCR2 expression with clinicopathological features in PCa patients.

Clinical features	TMA				TCGA			
	Case	Low, n (%)	High, n (%)	*P*	Case	Low, n (%)	High, n (%)	*P*
**Tissue**								
Cancer	70	6 (8.57)	64 (91.43)	0.260	427	213 (49.88)	214 (50.12)	–
Non-cancer	10	2 (20.00)	8 (80.00)	–	0	–	–	–
**Age (years)**								
≤60	14	2 (14.29)	12 (85.71)	0.556	190	107 (56.32)	83 (43.68)	0.017^*^
>60	66	6 (9.09)	60 (90.91)	–	237	106 (44.73)	131 (55.27)	–
**Gleason score**								
≤8	46	3 (6.52)	43 (93.48)	0.396	300	172 (57.33)	128 (42.67)	<0.001^***^
>8	24	3 (12.50)	21 (87.50)	–	127	41 (32.28)	86 (67.72)	–
**Serum PSA levels (ng/ml)**								
≤2	–	–	–	–	378	194 (51.32)	184 (48.68)	0.010^**^
>2	–	–	–	–	35	10 (28.57)	25 (71.43)	–
**Pathological grade**								
pT1-pT2	4	0 (0.00)	4 (100.00)	0.525	–	–	–	–
pT3	65	6 (9.23)	59 (90.77)	–	–	–	–	–
**Tumor stage**								
T1	0	–	–	**-**	153	88 (57.52)	61 (42.48)	0.053
T2	42	4 (9.52)	38 (90.48)	0.787	155	74 (47.74)	80 (52.26)	–
T3	14	1 (7.14)	13 (92.86)	–	48	22 (45.83)	32 (54.17)	–
T4	0	–	–	–	1	0 (0.00)	1 (100.00)	–
**Lymph nodemetastasis**								
N0	56	5 (8.93)	51 (91.07)	–	303	154 (50.83)	149 (49.17)	0.002^**^
N1	0	–	–	–	69	21 (30.43)	48 (69.57)	–
**Distant metastasis**								
M0	56	5 (8.93)	51 (91.07)	–	401	199 (49.63)	202 (50.37)	0.574
M1	0	–	–	–	3	1 (33.33)	2 (66.67)	–

＊means the difference is significant at the 0.05 level ,** means the difference is significant at the 0.01 level, *** means the difference is significant at the 0.01 level.

**Table 4:** Correlation of CCR7 expression with clinicopathological features in PCa patients.

Clinical features	TMA				TCGA			
	Case	Low, n (%)	High, n (%)	*P*	Case	Low, n (%)	High, n (%)	*P*
**Tissue**								
Cancer	70	31 (44.29)	39 (55.71)	0.734	427	213 (49.88)	214 (50.12)	–
Non-cancer	10	5 (50.00)	5 (50.00)	–	0	–	–	–
**Age (years)**								
≤60	14	7 (50.00)	7 (50.00)	0.679	190	84 (44.21)	106 (55.79)	0.036^*^
>60	66	29 (43.94)	37 (56.06)	–	237	129 (54.43)	108 (45.57)	–
**Gleason score**								
≤8	46	21 (45.65)	25 (54.35)	0.750	300	150 (50.00)	150 (50.00)	0.941
>8	24	10 (41.67)	14 (58.33)	–	127	63 (49.61)	64 (50.39)	–
**Serum PSA levels (ng/ml)**								
≤2	–	–	–	–	378	190 (50.26)	188 (49.74)	0.245
>2	–	–	–	–	35	14 (40.00)	21 (60.00)	–
**Pathological grade**								
pT1-pT2	4	2 (50.00)	2 (50.00)	0.834	–	–	–	–
pT3	65	29 (44.62)	36 (55.38)	–	–	–	–	–
**Tumor stage**								
T1	0	–	–	**-**	153	81 (52.94)	72 (47.06)	0.249
T2	42	17 (40.48)	25 (59.52)	0.277	155	69 (44.52)	86 (55.48)	–
T3	14	8 (57.14)	6 (42.86)	–	48	27 (56.25)	21 (43.75)	–
T4	0	–	–	–	1	0 (0.00)	1 (100.00)	–
**Lymph nodemetastasis**								
N0	56	25 (44.64)	31 (55.36)	–	303	147 (48.51)	156 (51.49)	0.450
N1	0	–	–	–	69	30 (43.48)	39 (56.52)	–
**Distant metastasis**								
M0	56	25 (44.64)	31 (55.36)	–	401	196 (48.88)	205 (51.12)	0.539
M1	0	–	–	–	3	2 (66.67)	1 (33.33)	–

*means the difference is significant at the 0.05 level.

**Table 5:** Correlation of CD28 expression with clinicopathological features in PCa patients.

Clinical features	TMA				TCGA			
	Case	Low, n (%)	High, n (%)	*P*	Case	Low, n (%)	High, n (%)	*P*
**Tissue**								
Cancer	70	35 (50.00)	35 (50.00)	0.554	427	213 (49.88)	214 (50.12)	–
Non-cancer	10	4 (40.00)	6 (60.00)	–	0	–	–	–
**Age (years)**								
≤60	14	9 (64.29)	5 (35.71)	0.200	190	98 (51.58)	92 (48.42)	0.530
>60	66	30 (45.45)	36 (54.55)	–	237	115 (48.52)	122 (51.48)	–
**Gleason score**								
≤8	46	25 (54.35)	21 (45.65)	0.314	300	156 (52.00)	144 (48.00)	0.179
>8	24	10 (41.67)	14 (58.33)	–	127	57 (44.88)	70 (55.12)	–
**Serum PSA levels (ng/ml)**								
≤2	–	–	–	–	378	193 (51.06)	185 (48.94)	0.026^*^
>2	–	–	–	–	35	11 (31.43)	24 (68.57)	–
**Pathological grade**								
pT1-pT2	4	1 (25.00)	3 (75.00)	0.317	–	–	–	–
pT3	65	33 (50.77)	32 (49.23)	–	–	–	–	–
**Tumor stage**								
T1	0	–	–	–	153	88 (57.52)	65 (42.48)	0.138
T2	42	23 (54.76)	19 (45.24)	0.272	155	76 (49.03)	79 (50.97)	–
T3	14	10 (71.43)	4 (28.57)	–	48	20 (41.67)	28 (58.33)	–
T4	0	–	–	–	1	0 (0.00)	1 (100.00)	–
**Lymph nodemetastasis**								
N0	56	33 (58.93)	23 (41.07)	–	303	148 (48.84)	155 (51.16)	0.215
N1	0	–	–	–	69	28 (40.58)	41 (59.42)	–
**Distant metastasis**								
M0	56	33 (58.93)	23 (41.07)	–	401	199 (49.63)	202 (50.37)	0.574
M1	0	–	–	–	3	1 (33.33)	2 (66.67)	–

＊means the difference is significant at the 0.05 level.

## Discussion

PCa is considered to be a multi-stage progressive disease, which generally develops from prostatic intraepithelial tumor to hormone dependent invasive adenocarcinoma in situ, and finally to hormone independent metastatic tumor. Radical treatments, such as radical prostatectomy, radiotherapy and cryotherapy, are always adopted for the early PCa. And for the advanced PCa, androgen deprivation therapy (ADT) is the main treatment for it, but most patients will gradually develop castration resistant PCa (CRPC). When the tumor infiltrates out of the prostate capsule, the treatment effect and prognosis become worse. At this time, the standard treatment is radiotherapy, followed by chemotherapy and enzalutamide, but some patients are prone to drug resistance (22). Docetaxel is the first drug approved by FDA to treat CRPC after ADT failure, which can significantly improve the survival rate of advanced PCa (23). Although enzalutamide and chemotherapeutic drugs can prolong the survival time of PCa patients, they will produce side effects such as drug resistance, and ultimately cannot curb tumor growth and metastasis. TME is filled with a large number of cancerous cells and stromal cells. The latter are usually composed of inflammatory cells, smooth muscle cells, adipocytes, fibroblasts, neuroendocrine cells, endothelial cells and microvessels, with cytokines, growth factors, low pH, hypoxia and various extracellular matrices (24). Many studies have found that (25–27), TME played an important role in the physiological regulation of inducing tumor acquired drug resistance and resisting apoptosis.

FCGRs is the receptor for the Fc segment of immunoglobulin (IgG), which can be divided into three types: FCGRI (CD64), FCGRII (CD32) and FCGRIII (CD16). The gene encoding FCGRs has polymorphism and is involved in the stimulation of many biological functions, such as phagocytosis, cell lysis and inflammatory cascade reactions (28–30). FCGR3A (CD16), a member of FCGRs family, is mainly expressed on the surface of natural killer (NK) cell membrane and is a transmembrane receptor, which plays a role of bridge for immune cells to directly kill target cells (31). HAVCR2 (Tim3), a member of Tim3 family, is expressed on the membrane of various immune cells as a transmembrane protein (32). HAVCR2 not only acts on differentiated and mature T lymphocytes, but also plays an immunomodulatory role in a variety of innate immune cells (33). Studies had found that HAVCR2 was highly expressed in NK cells in TME, and hepatocellular carcinoma cells could lead to dysfunction of NK cell population by inhibiting PI3K/Akt/mTORC1 signaling pathway mediated by HAVCR2 (34). Therefore, FCGR3A and HAVCR2 are highly expressed in various cancer models and are likely to participate in the immune process of NK cells. However, the roles of FCGR3A and HAVCR2 in PCa are rarely reported. We first analyzed the PPI network construction of 42 candidate genes, and found that FCGR3A and HAVCR2 interacted with each other, and they were closely related to CCR7 and CD28. As we konw, B7/CD28 is a classic costimulatory signaling pathway, and activation of this pathway can lead to activation of CD8^+^ and CD28^+^ T cells (35). However, most tumor cells can inhibit the activation of T cells by down-regulating which the expression of CD28 to achieve immune escape in TME (36). TME is also filled with a large number of inflammatory and chemotactic factors, which can activate tumor cells and promote their metastasis. It was found that TNF-α in TME could promote PCa metastasis and diffusion from lymph nodes by activating the CCL21/CCR7 signaling axis (37). Therefore, CCR7 and CD28 also play an important roles of immune regulation in TME.

During the occurrence and development of PCa, the relationship between FCGR3A, HAVCR2, CCR7 and CD28 was still unclear, which requires preliminary research. After incorporating this gene cluster into the prognostic model, we found that this gene cluster had the ability to predict the biochemical recurrence of PCa patients. Cox regression analysis showed that the risk ratio of high expression of these genes was 2.30 times higher than that of low expression in single-factor analysis, but there was no statistical significance in the multivariate analysis. We hypothesized that this might be due to the uneven expression of individual genes in the tissues of PCa patients. Subsequently, proof of our hypothesis by survival analysis and clinical tissue microarray assays. The results showed that the survival rate and prognosis of PCa patients were lower, and the high expression of FCGR3A and HAVCR2 proteins in PCa tissues increased gradually with the increase of GS when FCGR3A and HAVCR2 were overexpressed. These results were consistent with the findings of FCGR3A and HAVCR2 in other immunological or cancer diseases (38–41). These results suggest that the expression of FCGR3A and HAVCR2 is correlated with the degree of malignancy in PCa patients, and the high expression of FCGR3A and HAVCR2 has a poor prognosis for PCa patients. Finally, we compared the correlation between the expression of 4 target genes and different clinicopathological features of PCa patients. The results showed that the expression of FCGR3A had a unique correlation with the clinicopathological features of PCa. This is similar to FCGR3A in other tumor models, for example, the level of natural killing activity in peripheral blood mononuclear cells of patients with bladder cancer is correlated with the clinical evolution and pathological stage of the disease (42).

FCGR3A is the target of many drugs such as rituximab and its expression in prostate cancer cells is positively correlated with other markers (43). Long-term use of some common drugs leads to mutations in FCGR3A and related genes, which produces resistance to the disease, such as non-small cell lung cancer (44, 45). Different new disease targets, FCGR3A as an important marker gene for a variety of diseases, on the one hand, can promote the development of new uses of old drugs faster, on the other hand, through the analysis of the interaction between FCGR3A and other cancer markers, find out the dominant gene, which is conducive to the combined treatment of a variety of diseases. FCGR3A has developed resistance to some drugs, so it will be more difficult to develop new drugs.

In conclusion, FCGR3A is a biomarker with potential prognostic value for PCa, which can predict the survival of PCa patients and provide a new basis for rational administration in clinical of PCa patients.With the development of new technologies, FCGR3A is expected to become a new breakthrough point for potent drugs.

## Data availability statement

The original contributions presented in the study are included in the article/**Supplementary Material**. Further inquiries can be directed to the corresponding author.

## Ethics statement

Written informed consent was obtained from the individual(s) for the publication of any potentially identifiable images or data included in this article.

## Author contributions

ZZ and W-DZ contributed to conception and design of the study. YH organized the database. ZT performed the statistical analysis. Q-LD wrote the first draft of the manuscript. YW, S-BY, Y-DW, H-JT, and F-NJ wrote sections of the manuscript. All authors contributed to manuscript revision, read, and approved the submitted version.

## Funding

This work was supported by grants from National Natural Science Foundation of China (Grant No. 82072813, Grant No. 81571427), Scientific research start-up project of Guangzhou First People’s Hospital (Grant No. KYQD0004), Guangzhou General Science and Technology Project of Health and Family Planning (Grant No. 20201A011012), Guangzhou Planned Project of Science and Technology (Grant No. 202102010038, Grant No. 202102080029).

## Conflict of interest

The authors declare that the research was conducted in the absence of any commercial or financial relationships that could be construed as a potential conflict of interest.

## Publisher’s note

All claims expressed in this article are solely those of the authors and do not necessarily represent those of their affiliated organizations, or those of the publisher, the editors and the reviewers. Any product that may be evaluated in this article, or claim that may be made by its manufacturer, is not guaranteed or endorsed by the publisher.
